# Editorial: The influence of metal ions and their complexes on the function and structure of biological macromolecules

**DOI:** 10.3389/fchem.2025.1563537

**Published:** 2025-03-07

**Authors:** Miroslaw Gilski, Mannar R. Maurya, Boguslaw Nocek, Krzysztof Brzezinski

**Affiliations:** ^1^ Faculty of Chemistry, A. Mickiewicz University, Poznan, Poland; ^2^ Institute of Bioorganic Chemistry, Polish Academy of Sciences, Poznan, Poland; ^3^ Department of Chemistry, Indian Institute of Technology Roorkee, Roorkee, India; ^4^ Discovery Chemistry Research and Technology, Eli Lilly and Company, Lilly Corporate Center, Indianapolis, IN, United States

**Keywords:** enzyme activity, metal-based drugs, heavy-metal ions, inhibitors, intermolecular interactions

Metal cations play a critical role in cellular metabolism. They interact with various macromolecules, namely, proteins and nucleic acids, in every living cell, regulating and ensuring their biological functions. For instance, metal cations can activate or inhibit the catalytic activity of enzymes and RNA-based catalysts, such as ribozymes. Many non-catalytic proteins, including metalloproteins involved in gas transport, such as hemoglobin, myoglobin, and hemocyanin, require metal ions or their complexes to perform their biological functions. Certain cations interact with negatively charged DNA and RNA molecules, stabilizing structure and maintaining dynamics, and facilitating the storage, processing, and regulation of genetic information in cells. Not surprisingly, the concentrations of metal cations within a cell must be strictly controlled, as any disturbance, such as the introduction of toxic cations from the environment, can lead to numerous pathological processes or even cell death. Conversely, compounds that target metal-dependent macromolecules provide an excellent opportunity for drug intervention at the molecular level of cell metabolism. Therefore, the role and nature of interactions between metal ions or their complexes with proteins and nucleic acids is a major focus of current bioinorganic chemistry research.

This Research Topic describes diverse studies on interactions between macromolecules and metal ions or their complexes ranging from methodological aspects relevant to such research to structural and biochemical analyses of protein-metal ion complexes. The Research Topic includes five original research studies and two review articles.

Single-molecule techniques have revolutionized biophysical measurements, significantly simplifying experiments and allowing them to be performed with very low sample requirements. These techniques can characterize all types of biological macromolecules, namely, proteins, DNA and RNA. However, studies of macromolecules that bind metal cations are limited due to numerous limitations and obstacles. In their review, de la Torre and Pomorski focused on single-molecule methods, such as single-molecule FRET, nanopores and optical tweezers, that can be used to study macromolecules that bind metal cations. In particular, they discussed the potential promise and difficulties that could be experienced during single-molecule measurements.

Structural biology is a powerful tool for determining the three-dimensional structure of macromolecules of interest at atomic or near-atomic resolution. It allows the visualization of macromolecular interactions with bound ligands including metal cations. However, the interpretation of structural data is not always simple or obvious. The review by Majorek et al. highlighted issues related to the limitations of structural methods and erroneous interpretation of experimental data which can lead to misinterpretation of the role and function of the metal within the macromolecule. In particular, the authors highlighted various aspects of the structural data available for metal cation-protein complexes and examined the quality of modeling of metal ion binding sites across different structure determination methods.

The research by Canyelles i Font et al. underlined an issue related to biologically and environmentally ubiquitous metal cations in the context of affecting high-throughput screening (HTS) enzyme-based assays. Indeed, contamination of chemical libraries, buffers, biological samples and more with cations that interfere with assay components can generate significant errors and lead to incorrect conclusions. The authors tested three luciferase variants commonly used in bioluminescent HTS assays and analyzed the impact of metal ions on luciferase-mediated bioluminescence. The study revealed significant quenching effects over biologically and environmentally relevant concentration ranges of metal ions indicating a substantial influence on HTS assays and the subsequent interpretation of experimental data. Based on the results obtained, the authors proposed a series of strategies that would allow the selection and modification of an assay best suited to the type of metal cation contamination.

Ruthenium complexes are considered diagnostic and therapeutic agents that target a variety of protein molecules. The study by Oszajca et al. presented crystallographic and physicochemical analyses of several Ru(III) complexes-hen-egg white lysozyme adducts. The obtained results provided insights into the structure and stability of the adducts, suggesting how the nature of the *N*-heterocyclic ligands and their hydrolytic behavior influence the binding of Ru(III) complexes to proteins.

Finally, three original studies described the biochemical and structural research of bacterial enzymes in the context of the role of zinc ions in catalysis. The research by Kelley et al. detailed structural and biochemical studies of *N* ^α^-acetyl-L-ornithine deacetylase from *Escherichia coli*, a promising antibiotic drug target. The authors reported two crystal structures of this enzyme containing one or two zinc ions coordinated within the active site area. Two publications from Mariusz Jaskolski’s group focused mainly on the interactions of bacterial L-asparaginases from *Rhizobium etli* with zinc ions. The research by Sliwiak et al. showed that two L-asparaginase isoforms, ReAIV and ReAV, have different biochemical characteristics, complementing these forms under distinct environmental and physiological conditions. In particular, these two isoforms revealed a different response to various transition metal cations such as zinc. Finally, structural and biochemical studies of ReAV, and a mutagenic analysis of this enzyme described by Pokrywka et al., suggested a role of zinc cations in ReAV activity.

The original research and review articles published in this Research Topic offered insights into the current state of bioinorganic chemistry, notably from various perspectives. Obviously, structural studies of biological macromolecular complexes with metal cations are of key importance for bioinorganic research. On the other hand, there is still room for the development of other biophysical methods that could be applied to analyze the influence of metal ions and their complexes on the function and structure of biological macromolecules. We hope that this Research Topic will stimulate further research, advancing our understanding of macromolecule-metal cation interactions and their implications for human health and disease.

